# Supplementary material for: Single-Inclusion Kinetics of Chlamydia trachomatis Development
Source: mSystems. 2020 Oct 13;5(5):e00689-20. doi: 10.1128/mSystems.00689-20 (PMC7567582; doi:10.1128/mSystems.00689-20)
Supplement: FIG S1 [file mSystems.00689-20-sf001.pdf]

Supplemental Information: Description of Mathematical Models

Model Description

We developed two systems of ordinary differential equations to describe the temporal dynamics of reticulate body (RB) to elmentary body (EB) conversion. Both models track RB (R) replication, their conversion to intermediate bodies (IB, I), and subsequent conversion to EBs (E). In both models, a signal (S) acts as an inhibitor of RB to IB conversion. The models differ in the location of the signal. In the *environmental signal model*, the signal is located outside of the RB and acts globally on the entire population of *Chlamydia* while in the *intrinsic signal model* the signal is located within each individual RB.

In both models, we assume that cells grow exponentially at per capita rate r. The rate of RB conversion to IB follows an inverse Hill function that depends on the concentration of the signal S. IBs convert to EBs at per capita rate δ. The signal S is depleted by RBs R at a rate proportional to the RB population. In a single population of cells (multiplicity of infection = 1), this leads to the following system of differential equations for both models

$$\begin{aligned}
\frac{dR}{dt} &= rR - \frac{\beta R}{(S/\kappa)^n + 1} \\
\frac{dI}{dt} &= \frac{\beta R}{(S/\kappa)^n + 1} - \delta I \\
\frac{dE}{dt} &= \delta I \\
\frac{dS}{dt} &= -\gamma S R.
\end{aligned}$$

Parameters for the model are given in Table 1.

Parameter	Description	Value
r	RB growth rate	0.31 hour^{-1}
β	maximum per capita RB to IB conversion rate	2.5 hour^{-1}
n	Hill coefficient	2
κ	Half-saturation constant	$1/10S_0$
δ	per capita IB to EB conversion rate	1 hour^{-1}
γ	Signal depletion rate	0.005 cell^{-1} hour^{-1}

Table 1: Description of parameters used in the mathematical models.

Multiple infections and superinfections

The models differ if host cells are multiply infected or superinfected. In each of these cases, the RBs can be divided into subpopulations R_i for $i = 1, ..., m$ that represent RBs derived from each infection. As previously

mentioned, in the environmental signal model the signal S is shared by the entire RB population. As such, the governing equations are

$$\begin{aligned}
\frac{dR_i}{dt} &= rR_i - \frac{\beta R_i}{(S/\kappa)^n + 1} \\
\frac{dI_i}{dt} &= \frac{\beta R_i}{(S/\kappa)^n + 1} - \delta I_i \\
\frac{dE_i}{dt} &= \delta I_i \\
\frac{dS}{dt} &= -\gamma S \sum_{i=1}^{m} R_i.
\end{aligned} \tag{1}$$

In contrast, in the intrinsic model the signal S is unique to each RB so that the governing equations are

$$\begin{aligned}
\frac{dR_i}{dt} &= rR_i - \frac{\beta R_i}{(S_i/\kappa)^n + 1} \\
\frac{dI_i}{dt} &= \frac{\beta R_i}{(S_i/\kappa)^n + 1} - \delta I_i \\
\frac{dE_i}{dt} &= \delta I_i \\
\frac{dS_i}{dt} &= -\gamma S_i R_i.
\end{aligned} \tag{2}$$

Differentiating between the environmental and intrinsic models

To determine conditions in which the temporal dynamics of RBs and EBs differ in the environmental and intrinsic models, we numerically solved models (1) and (2) using the `odeint` function from the `SciPy` package under a variety of scenarios. Specifically, we considered variations in multiplicity of infection (MOI), growth rate, and superinfection.

Parameters and initial conditions for base model

Numerical solutions were initiated at 8 hours post infection (HPI) and continued until 48 HPI. The RB generation time was calculated to be at max 2.27 hrs as measured by the increase in *ihtA*prom-EGFP fluorescence using the Growthcurver package in R (47).

By definition, MOI = 1 equates to an average of one EB infecting each host cell. This scenario was used as the baseline for both the environmental and intrinsic models (eqns. (1) and (2)). For these systems of differential equations an MOI = 1 translates into an initial RB population of 1, and as cell-type conversion has yet occur, the initial IB and EB populations are set to 0.

Growth rate

To test the effect of growth rate on each model we implemented the initial conditions from the MOI = 1 scenario, but varied the RB generation time (r).

MOI

To test the effect of MOI on each model, the initial RB, IB, and EB cell-types were divided into 10 subpopulations. The simulations differed in that the environmental model contained a global signal concentration

S shared by all RB subpopulations (eqns. (1)), where the intrinsic model contained 10 independent signal subpopulations S_i which corresponded to each RB subpopulation (eqns. (2)).

Superinfection

To test the effect of superinfection on each model we devised two scenarios. For the environmental model (eqns. (1)) we simulated a primary infection at an MOI = 1 saving the 18 HPI RBs, IBs, EBs, and signal concentration values. We then simulated a secondary infection (MOI = 1) and used the 18 HPI signal concentration as S_0. The primary infection RB, IB, and EB values were input as a subpopulation in the secondary infection. For the intrinsic model (eqns. (2)), the secondary infection was introduced, but with a self contained signal concentration independent of the primary infection.
