# Supplementary figures and images for: Single-Inclusion Kinetics of Chlamydia trachomatis Development
Source: mSystems. 2020 Oct 13;5(5):e00689-20. doi: 10.1128/mSystems.00689-20 (PMC7567582; doi:10.1128/mSystems.00689-20)

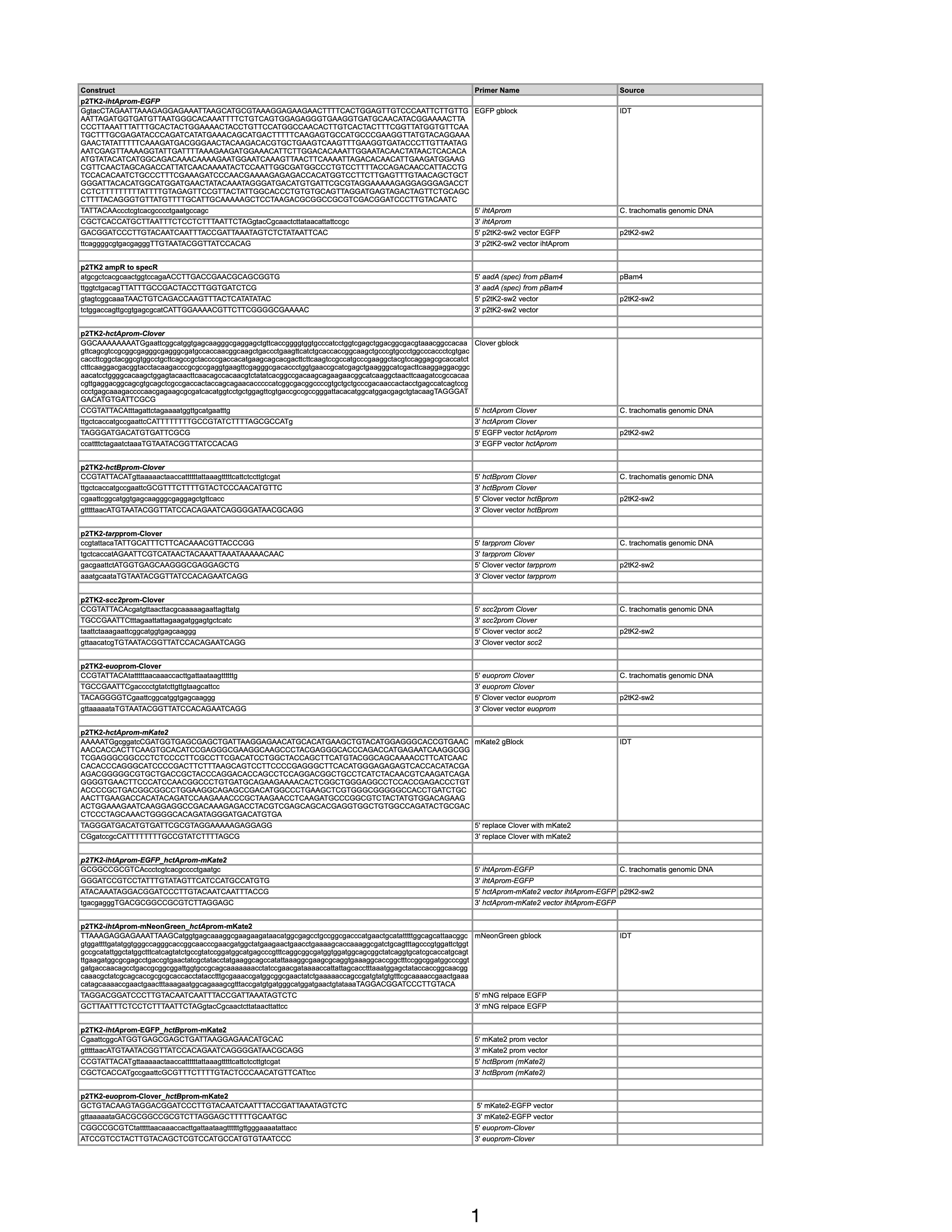

Supplement: Table S1 [file mSystems.00689-20-st001.tif]

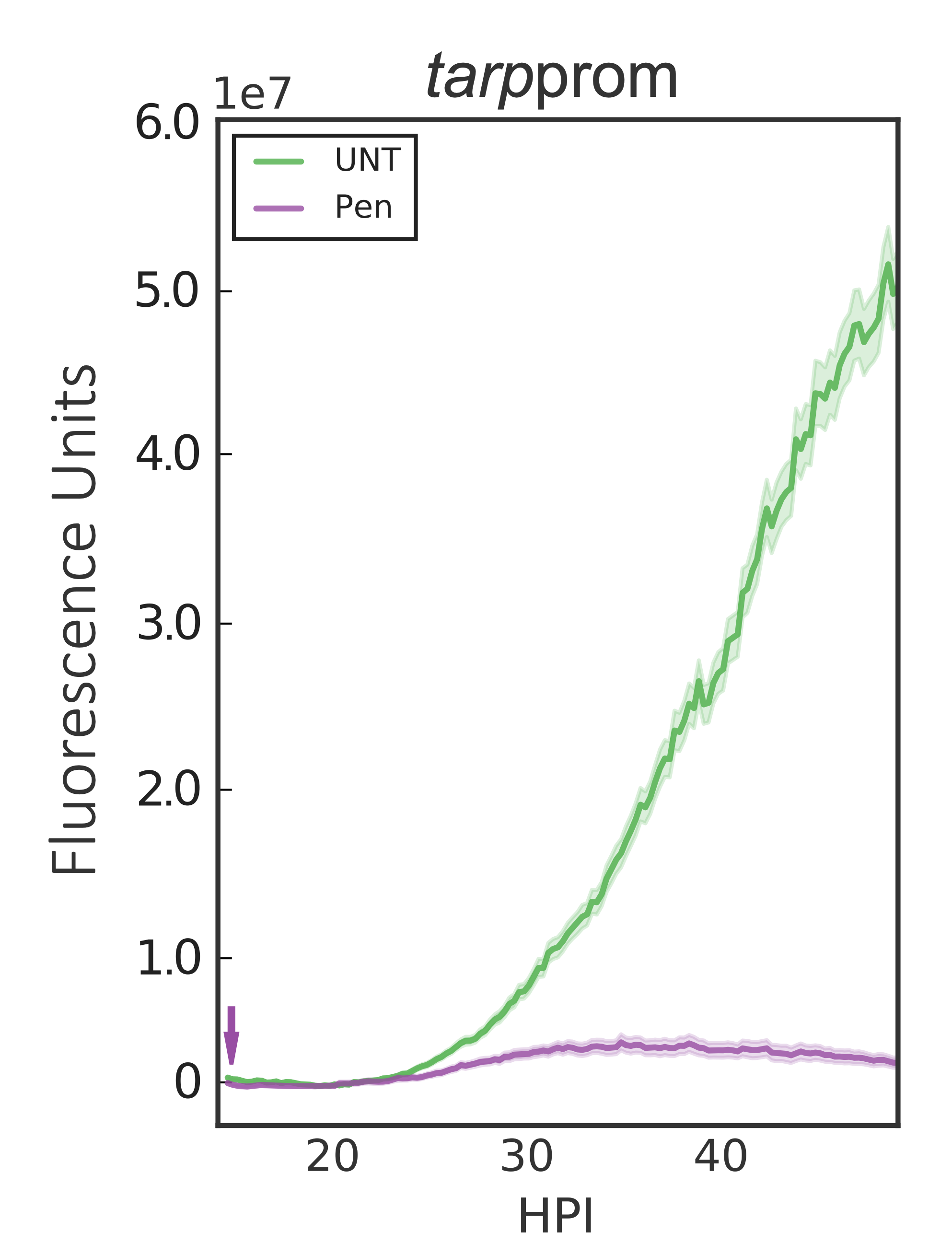

Supplement: FIG S2 [file mSystems.00689-20-sf002.tif]

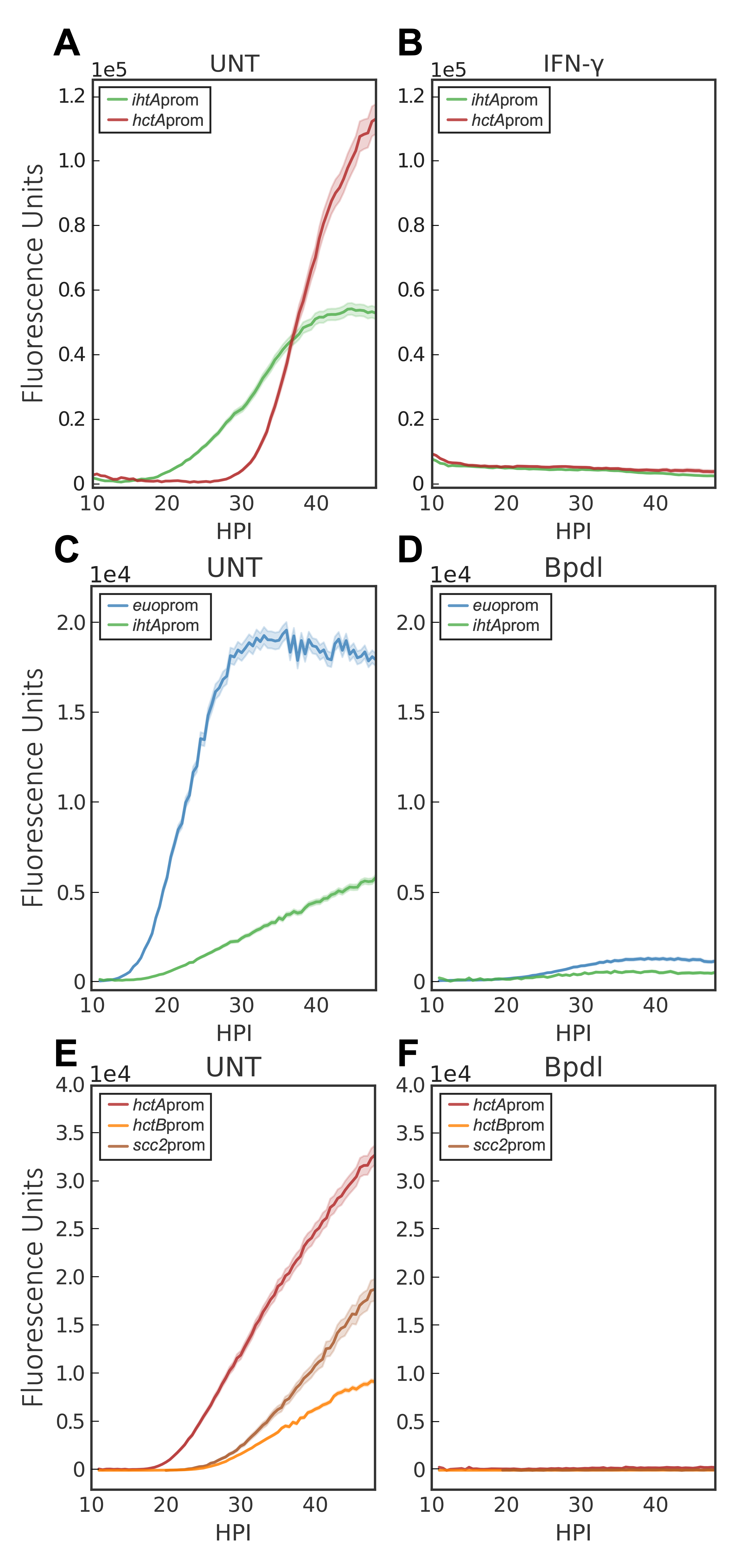

Supplement: FIG S3 [file mSystems.00689-20-sf003.tif]

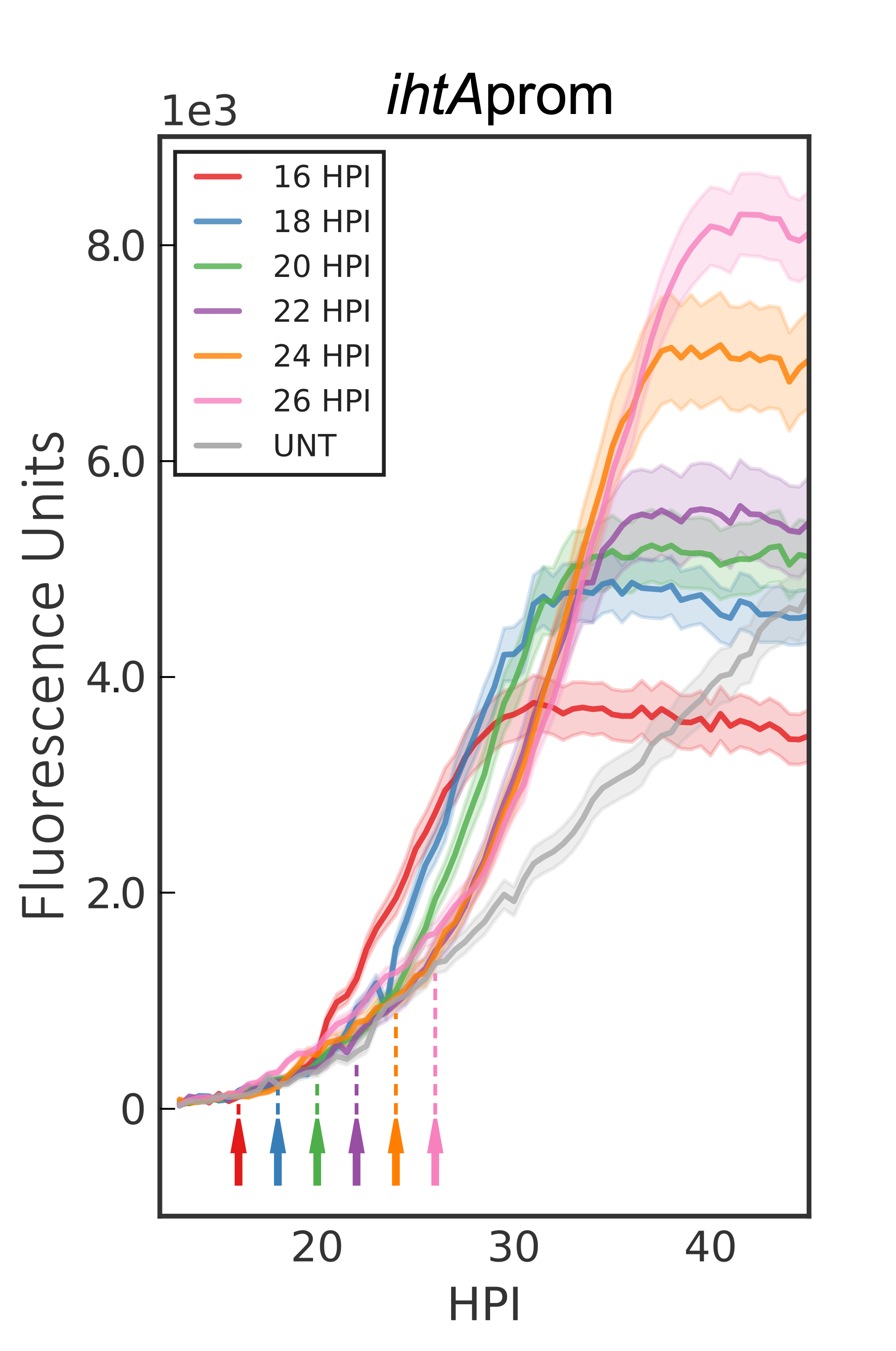

Supplement: FIG S4 [file mSystems.00689-20-sf004.tif]

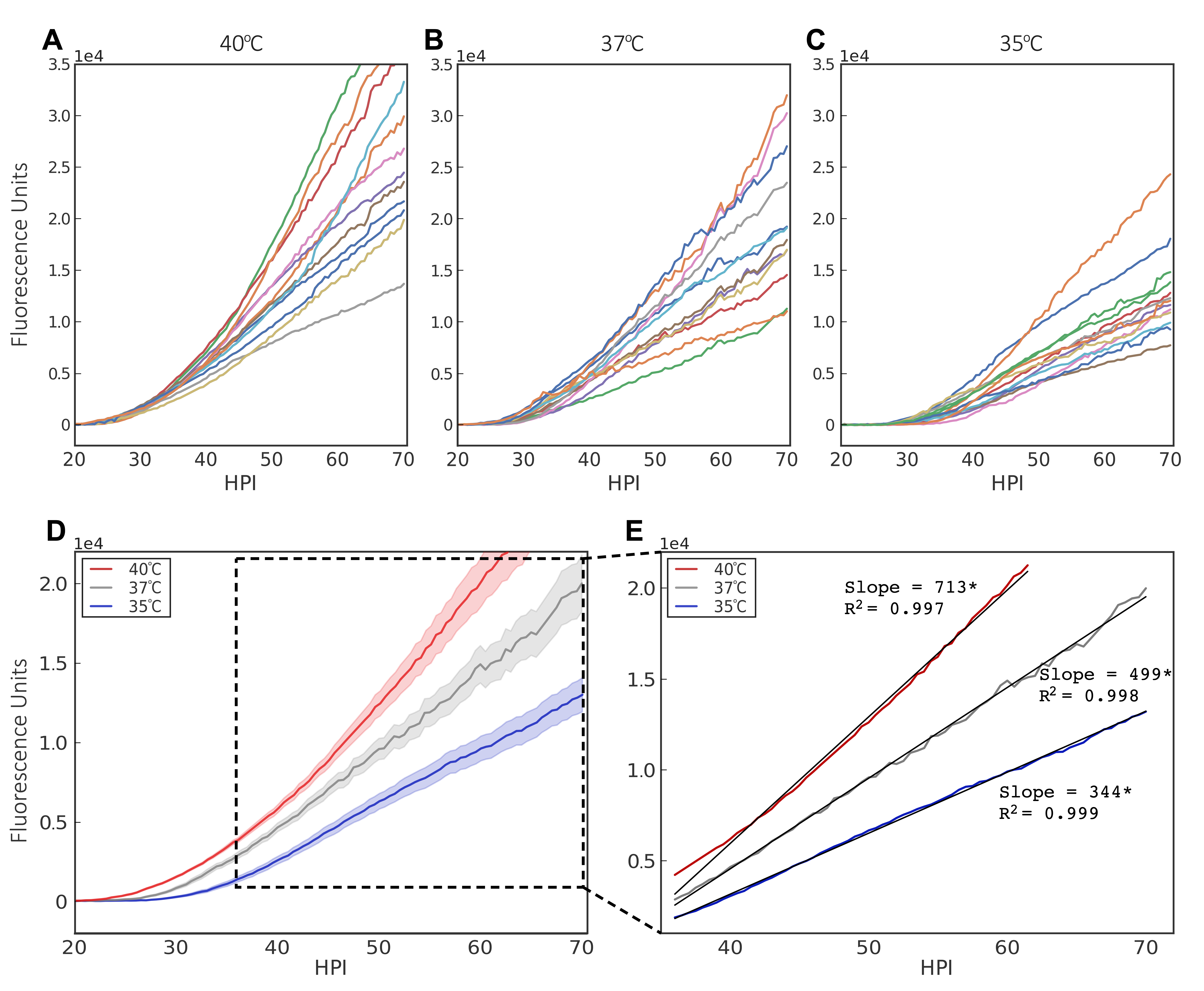

Supplement: FIG S5 [file mSystems.00689-20-sf005.tif]
